# Supplementary material for: The interactome of intact mitochondria by cross-linking mass spectrometry provides evidence for coexisting respiratory supercomplexes
Source: Mol Cell Proteomics. 2017 Dec 8;17(2):216–32. doi: 10.1074/mcp.RA117.000470 (PMC5795388; doi:10.1074/mcp.RA117.000470)
Supplement: Supplemental Data [file supp_RA117.000470_133922_0_supp_23978_fzffwf.zip › spectra_annotation/mito_DR_spectra_annotation/197-2-6-1-5-1.pdf]

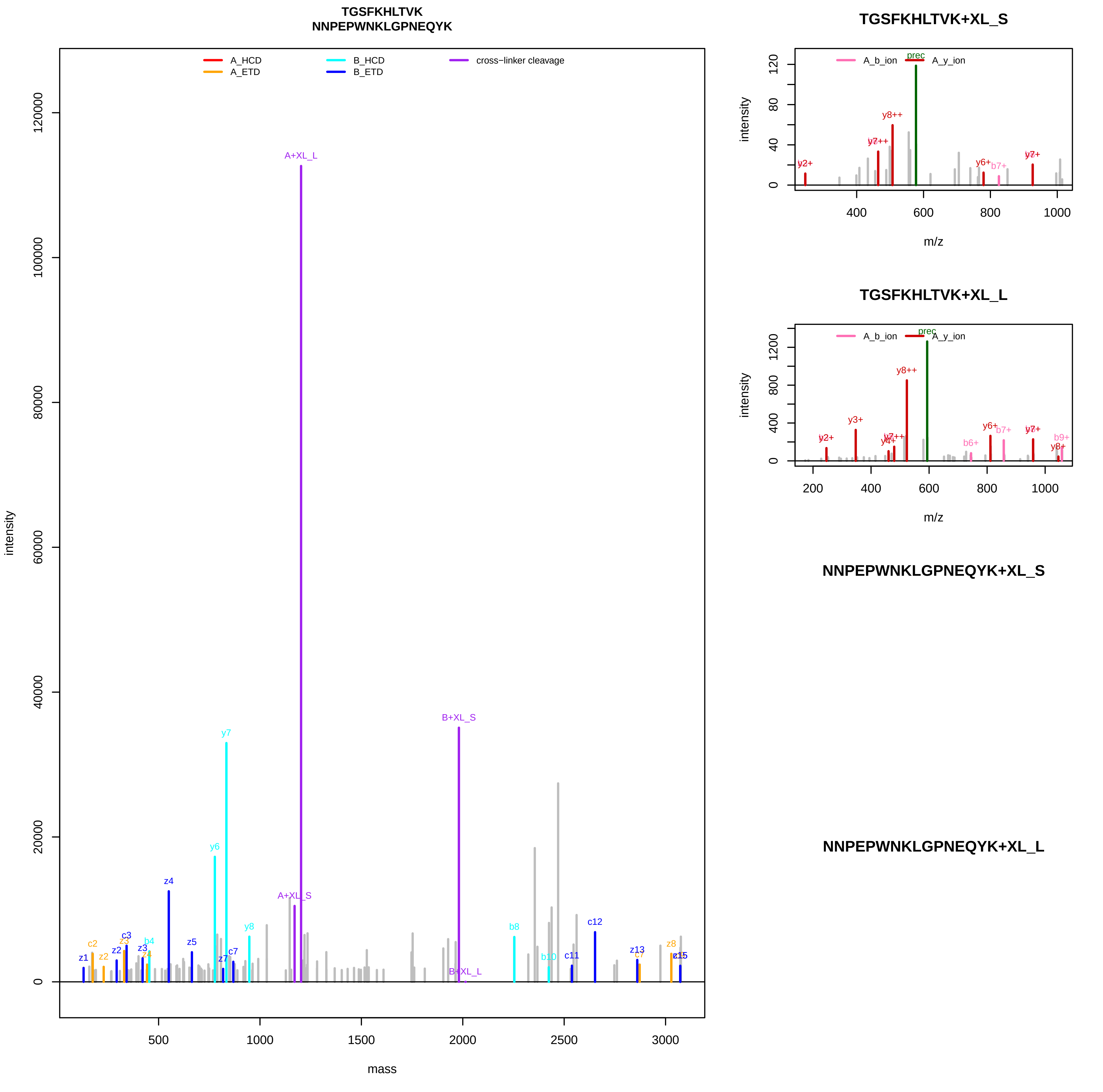

TGSFKHLTVK
NNPEPWNKLGPNEQYK
A_HCD
A_ETD
B_HCD
B_ETD
cross−linker cleavage
intensity
mass
A+XL_L
A+XL_S
B+XL_S
B+XL_L
y6
y7
y8
b4
b8
b10
z1
z2
z3
z4
z5
z7
z8
z13
z15
c2
c3
c7
c11
c12
c15
TGSFKHLTVK+XL_S
TGSFKHLTVK+XL_L
A_b_ion
A_y_ion
prec
m/z
y2+
y3+
y4+
y6+
y7+
y8+
y7++
y8++
b6+
b7+
b9+
NNPEPWNKLGPNEQYK+XL_S
NNPEPWNKLGPNEQYK+XL_L
